# Combinations of self‐reported rhinitis, conjunctivitis, and asthma predicts IgE sensitization in more than 25,000 Danes

**DOI:** 10.1002/clt2.12013

**Published:** 2021-03-30

**Authors:** Susan Mikkelsen, Khoa Manh Dinh, Jens Kjærgaard Boldsen, Ole Birger Pedersen, Gitte Juel Holst, Mikkel Steen Petersen, Kathrine Agergård Kaspersen, Bjarne Kuno Møller, Kaspar Rene Nielsen, Helene Martina Paarup, Klaus Rostgaard, Henrik Hjalgrim, Erik Sørensen, Linda Jenny Handgaard, Thomas Folkmann Hansen, Karina Banasik, Kristoffer Sølvsten Burgdorf, Henrik Ullum, Torben Sigsgaard, Christian Erikstrup

**Affiliations:** ^1^ Department of Clinical Immunology Aarhus University Hospital Aarhus Denmark; ^2^ Danish Big Data Centre for Environment and Health (BERTHA) Aarhus University Roskilde Denmark; ^3^ Department of Clinical Immunology Zealand University Hospital Køge Denmark; ^4^ The Danish Clinical Quality Program–National Clinical Registries (RKKP) Central Denmark Region Aarhus Denmark; ^5^ Department of Clinical Immunology Aalborg University Hospital Aalborg Denmark; ^6^ Department of Clinical Immunology Odense University Hospital Odense Denmark; ^7^ Department of Epidemiology Research Statens Serum Institut Copenhagen Denmark; ^8^ Department of Clinical Immunology Copenhagen University Hospital Copenhagen Denmark; ^9^ Department of Neurology Danish Headache Center Copenhagen University Hospital Glostrup Denmark; ^10^ Faculty of Health and Medical Sciences Novo Nordisk Foundation Center for Protein Research University of Copenhagen Copenhagen Denmark; ^11^ Statens Serum Institut Copenhagen Denmark; ^12^ Department of Public Health Aarhus University Aarhus Denmark

**Keywords:** allergic IgE sensitization, asthma, conjunctivitis, environment, rhinitis

## Abstract

**Background:**

Allergic rhinitis (AR), allergic conjunctivitis (AC), and asthma composing multiple phenotypes and improved understanding of these phenotypes and their respective risk factors are needed.

**Objectives:**

The objective of this study was to define the prevalence of AR, AC, and asthma and their association with allergen‐specific immunoglobulin E (sIgE) sensitization in a large cohort of blood donors and identify risk factors.

**Methods:**

From the nationwide population‐based Danish Blood Donor Study, 52,976 participants completed an electronic questionnaire including AR, AC, asthma, allergic predisposition, and childhood residence. Of these, 25,257 were additionally tested for sIgE to inhalation allergens (Phadiatop).

**Results:**

The prevalence of sIgE sensitization, AR, AC, and asthma was 30%, 19%, 15%, and 9%, respectively. The youngest birth cohorts had the highest prevalence of sIgE sensitization and symptoms of asthma, AR, and AC, and for asthma, they apparently experienced symptoms at an earlier age. The sIgE sensitization was positively associated with male sex. The sIgE seroprevalence was higher in participants with both AR and AC (ARC) than in participants with either AR or AC. Allergic predisposition and sIgE sensitization increased the risk of the diseases, while farm upbringing was associated with reduced prevalence of ARC, however, only in sIgE sensitized participants.

**Conclusion:**

Birth year, childhood residence, sIgE sensitization, and allergic predisposition were associated with asthma, AR, and AC prevalence. Individuals with self‐reported ARC represent a primarily sIgE‐positive phenotype, while those with either AR or AC represent more diverse phenotypes.

## 1 INTRODUCTION

Allergic rhino‐conjunctivitis and asthma are common chronic diseases in the adult population with a prevalence of 20%–26% and 7%–11%, respectively.^1^, ^2^, ^3^ Prevalence of the diseases has increased over the last 20 years,^4^ and they negatively affect the quality of life^5^, ^6^ and performance in school and at work.^7^, ^8^ In allergic rhinitis (AR) and allergic conjunctivitis (AC), immunoglobulin E (IgE) is a central mediator of allergic inflammation induced by allergen inhalation. Allergic sensitization is characterized by the presence of allergen‐specific IgE (sIgE). The prevalence of inhalant allergen sensitization is 19%–50% in the general population.^9^, ^10^ Inhalant allergen sensitization is not always concordant with allergic symptoms.^11^, ^12^ Rhinitis and conjunctivitis may be either allergic or non‐allergic.

AR, AC, and asthma are heterogonous diseases with interplay between environmental and genetic factors, and they commonly coexist in individuals (multimorbidity) and within families. Children with a parental history of atopic diseases develop symptoms more frequently and at a younger age compared with children of non‐allergic parents.^13^ Obesity,^14^, ^15^ smoking,^16^, ^17^ and exposure to airborne allergens and pollution^18^, ^19^, ^20^ are associated with AR, AC, and asthma. Living on farms in early childhood (an environment rich in microbial compounds^21^), on the other hand, has a protective effect that persists in adulthood.^2^, ^10^, ^22^

The overall aim of this study was to describe AR, AC, asthma, and inhalant allergen sensitization in a Danish nationwide cohort of adult blood donors. The specific aims were to (1) determine the prevalence of AR, AC, asthma, and allergen sensitization; (2) explore associations between birth year and AR, AC, and asthma in single disease and multimorbidity with regards to prevalence and debut of symptoms; (3) explore associations between allergen sensitization, and AR, AC, and asthma; (4) assess potential risk factors for AR, AC, asthma, and sensitization; (5) examine associations between geographical upbringing and AR, AC, asthma, and sensitization; and (6) describe months with symptoms of allergy and/or asthma in different phenotypes.

Individuals must be in good overall health to be accepted as blood donors, and we thus expected slightly lower disease prevalence than in the general adult population, with the lowest prevalence in the oldest donors. We hypothesized that sIgE sensitization, allergic predisposition, age, and obesity were associated with increased risk for AR, AC, and asthma, whereas farm upbringing and smoking would have a protective effect. To our knowledge, this is one of the largest studies with data on AR, AC, and asthma in relation to inhalant allergen sensitization.

## 2 METHODS

### 2.1 Participants

Study participants consented to participate in the Danish Blood Donor Study (DBDS), a nationwide prospective, population‐based study and bio‐bank established in 2010.^23^, ^24^, ^25^ The participants are healthy adult blood donors aged 18–67 years. Blood donors with AR and AC are permitted to donate blood if they are asymptomatic on the day of donation without taking antihistamines during the last 24 h. Asthmatic donors are allowed to donate blood if they are asymptomatic on inhalation treatment at the time of donation. In Denmark, individuals with suspected or confirmed allergy to medication, latex, food, or insect bites are not accepted as blood donors.

### 2.2 Questionnaire and registers

From May 2015 to May 2018, a total of 52,976 consenting blood donors completed an electronic questionnaire while donating blood.^26^ Questions regarding allergy, asthma, and place of upbringing were based on standardized questions from the European Community Respiratory Health Survey (ECRHS) questionnaire.^27^ AR was defined as an affirmative answer to the question: “Do you have any nasal allergies including hay fever?”, AC as an affirmative answer to the question: “Do you have any eye allergies including hay fever?”, and ARC as both AR and AC. Asthma was defined as an affirmative answer to the question: “Do you have or have you ever had asthma?”. Allergic and asthmatic participants stated their age at the time of the first symptoms (in 5–10‐year age intervals) and months with symptoms.

**FIGURE A1:**
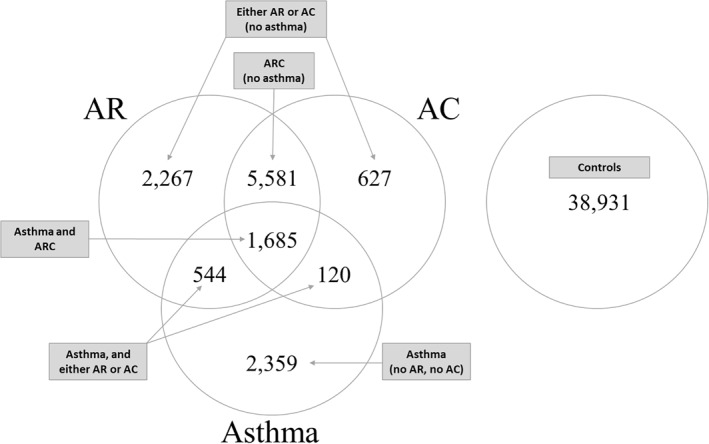
Distribution of phenotypes. Distribution of self‐reported allergic rhinitis (AR), allergic conjunctivitis (AC), asthma, and the six phenotype groups in the overall study population. Irrespective of sex. Controls: no AR, no AC, and no asthma. Results are presented as numbers. In total 862 (1.6%) could not be categorized due to missing data in self‐reported AR, AC, or asthma

Based on questionnaire data, the study population was divided into distinct single disease and multimorbidity groups (the coexisting of asthma, AR, and/or AC). However, “only AR” and “only AC” were merged for most analyses to avoid low numbers and insufficient power in these groups. This resulted in six distinct phenotype groups: asthma without AR and without AC; asthma with either AR or AC; asthma with ARC; ARC without asthma; either AR or AC without asthma; and controls (no AR, no AC, and no asthma). For distribution of phenotypes, see Figure A1.

Body mass index (BMI) (kg/m^2^) was calculated from self‐reported weight and height, and obesity was defined as BMI ≥ 30 kg/m^2^.^28^ Place of upbringing included six categories of urbanization. Smoking behavior included current, former, and never smoker. More details about the questionnaire and definitions are available in Appendices, Questionnaire.

All citizens in Denmark are assigned a unique 10‐digit personal identification number (civil registration number), which allows accurate linkage between individuals and register data. Data on age, sex, and current municipality residence were retrieved from Danish registers. Current municipality residence was divided into small (less than 100,000 citizens) and large density populated municipality (at least 100,000 citizens).

Current age was divided into 10‐year age strata or two age groups (younger than 45 and 45 years or older). This upper age limit is normally set to exclude participants with chronic obstructive pulmonary disease, which is uncommon before the age of 45.^1^, ^2^, ^29^

### 2.3 Serological testing

All blood donors included in DBDS from May 2015 to March 2017 in the Central Denmark Region, Capital Region of Denmark, Region Zealand, and North Denmark Region (*N* = 25,257) were tested for IgE antibodies specific for the nine most common respiratory sensitizers by a commercially available enzyme‐linked immunosorbent assay (ImmunoCAP Phadiatop; Phadia) at Thermo Fisher Scientific. The assay included the allergens: common silver birch (t3), Timothy grass (g6), mugwort (w6), cat dander (e1), dog dander (e5), horse dander (e3), house dust mites (*Dermatophagoides pteronyssinus* [d1] and *Dermatophagoides farinae* [d2]), and molds (*Penicillium chrysogenum* [m1], *Cladosporium herbarum* [m2], *Aspergillus fumigatus* [m3], and *Alternaria alternata* [m6]). The test result was considered positive if the concentration of sIgE was ≥0.35 kU/L.^30^

EDTA gel separated blood samples were centrifuged and stored at −20°C prior to testing. The blood‐sampling date corresponded to the date of the questionnaire response.

### 2.4 Statistical analysis

In the following, we define the prevalence of AR, AC, and asthma as the percentage who are currently or have a history of suffering from these conditions based on self‐reported data.

Statistical analysis was performed in Stata/MP 16.1, RStudio 1.2, and R 3.6.0. Results were reported as percentages with 95% confidence intervals (CIs), and medians with interquartile ranges (IQRs). Groups were compared by Mann–Whitney *U* test or Kruskal–Wallis test for non‐normally distributed data, and chi‐squared test for categorical data whenever all estimated counts were >5, and a 10^5^‐fold Monte Carlo simulated estimate for Fisher's exact test whenever some estimated counts were ≤5. Predictors of risk were analyzed by multivariable logistic regression analysis (if two outcomes) and presented as odds ratios (ORs) with 95% CIs, or multinomial logistic regression analysis (if more than two outcomes) and presented as relative risk ratios (RRRs, the ratio between two risk estimates) with 95% CIs.

The association between time of birth (10‐year birth cohorts) and age at the time of first symptom of asthma, AR, and AC in distinct single diseases and multimorbidity as well as in overlapping symptom groups were presented as cumulative incidence proportion and also analyzed by Poisson regression analysis. Participants entered the study at the date of birth and were right censored on the date of inclusion in the DBDS if they remained asymptomatic. Interval‐censored outcomes were constructed for participants experiencing symptoms of asthma, AR, and AC before inclusion. For multimorbidity groups, age at the time of first symptom of any of the current multimorbidities was used. In addition, the effect of the number of blood donations (1–10, 11–20, >20) on the association between birth year and age at the time of first symptom of asthma, AR, and AC were explored as a measure of survival bias. Results were presented as hazard ratios (HRs) with 95% CIs.

We tested for interactions in the Poisson and logistic regression models. If sex modified the effects of the exposures on the outcome, analyses were stratified by sex; otherwise, overall pooled estimates of the effects adjusted by sex were calculated.

The association between different Phadiatop cutoff values and self‐reported ARC was analyzed by receiver operating characteristic (ROC) curve.

### 2.5 Ethics

The DBDS was approved by the Central Denmark Region Ethics Committee (M‐20090237). The biobank and research database were approved by the Danish Data Protection Agency (P‐2019‐99). Oral and written informed consent was obtained from all participants.

## 3 RESULTS

For demographics of the study population stratified by sex, see Table 1. Differences between males and females were present for allergic predisposition, current age, BMI, smoking behavior, place of upbringing, and the distribution between allergy and asthma phenotype groups.

**TABLE 1:** Demographic of the study population stratified by sex (*N* = 52,976)

*N* (%)			Male 27,235 (51.4)	Female 25,741 (48.6)	Comparison	Total 52,976 (100)
		N	Median (IQR)	Median (IQR)		Median (IQR)
Age		52,976	41.2 (29.9–51.6)	39.3 (26.7–50.6)	**	40.3 (28.3–51.1)
BMI		52,824	25.5 (23.5–28.0)	24.4 (22.1–27.6)	**	25.0 (22.9–27.8)

		N	% (95% CI)	% (95% CI)		% (95% CI)
Current smokers		7008	13.0 (12.6–13.4)	13.7 (13.3–14.2)	*	13.4 (13.1–13.7)
Former smokers		11,884	21.6 (21.1–22.1)	23.3 (22.8–23.8)	**	22.4 (22.1–22.8)
Large municipality		20,665	38.6 (38.0–39.2)	39.5 (38.9–40.0)	NS	39.0 (38.6–39.4)
Obesity		7204	12.7 (12.3–13.1)	14.6 (14.2–15.0)	**	13.6 (13.3–13.9)
Parental allergy		9229	15.5 (15.1–15.9)	19.5 (19.0–20.0)	**	17.5 (17.1–17.8)
Age strata (years)						
18–24		8219	12.1 (11.7–12.5)	19.1 (18.7–19.6)		15.5 (15.2–15.8)
25–34		12,395	24.1 (23.6–24.6)	22.7 (22.2–23.2)		23.4 (23.0–23.8)
35–44		11,531	22.9 (22.4–23.4)	20.6 (20.1–21.1)	**	21.8 (21.4–22.1)
45–54		12,138	23.6 (23.1–24.1)	22.1 (21.6–22.7)		22.9 (22.6–23.3)
55–67		8693	17.3 (16.9–17.8)	15.4 (15.0–15.9)		16.4 (16.1–16.7)
Place of upbringing						
Farm with livestock		7764	13.9 (13.5–14.3)	15.6 (15.1–16.0)		14.7 (14.4–15.0)
Farm without livestock		1222	2.46 (2.28–2.65)	2.16 (1.99–2.35)		2.32 (2.19–2.45)
Rural area		10,075	19.0 (18.6–19.5)	19.2 (18.7–19.6)	**	19.1 (18.8–19.4)
Small town		17,333	32.8 (32.3–33.4)	32.9 (32.3–33.5)		32.8 (32.4–33.2)
Suburb		10,372	20.1 (19.6–20.5)	19.2 (18.8–19.7)		19.7 (19.3–20.0)
Inner city		6006	11.8 (11.4–12.2)	11.0 (10.6–11.4)		11.8 (11.1–11.7)
Phenotype groups						
1	Controls	38,931	74.0 (73.4–74.5)	75.5 (75.0–76.0)		74.7 (74.3–75.1)
2	Asthma (no AR, no AC)	2359	4.35 (4.11–4.60)	4.72 (4.46–4.98)		4.53 (4.35–4.71)
3	Asthma and AR (no AC)	544	1.09 (0.97–1.22)	1.00 (0.88–1.13)		1.04 (0.96–1.13)
3	Asthma and AC (no AR)	120	0.22 (0.17–0.28)	0.25 (0.19–0.31)		0.23 (0.19–0.28)
4	Asthma and ARC	1685	3.46 (3.25–3.69)	2.99 (2.79–3.21)	**	3.23 (3.08–3.39)
5	ARC (no asthma)	5581	11.3 (10.9–11.7)	10.1 (9.70–10.4)		10.7 (10.4–11.0)
6	AR (no asthma, no AC)	2267	4.54 (4.30–4.79)	4.15 (3.91–4.40)		4.35 (4.18–4.53)
6	AC (no asthma, no AR)	627	1.06 (0.94–1.19)	1.35 (1.22–1.50)		1.20 (1.11–1.30)

*Notes:* Results are presented as percentages with corresponding 95% CI and medians with IQR. Groups were compared by Mann–Whitney *U* test for non‐normally distributed data and chi‐squared test for categorical data. *p*‐Values were Bonferroni corrected; Obesity: BMI ≥ 30 kg/m^2^; Numbers 1–6 indicates the six distinct phenotype groups.

Abbreviations: AC, allergic conjunctivitis; AR, allergic rhinitis; ARC, both AR and AC; CI, confidence intervals; IQR, interquartile ranges; NS, non‐significant.

^*^ *p* < 0.05.

^**^ *p* < 0.001.

### 3.1 Prevalence of AR, AC, asthma, and inhalant allergen sensitization

The overall prevalence of self‐reported AR, AC, and asthma among 52,976 blood donors was 19%, 15%, and 9%, respectively. The prevalence of inhalant allergen sensitization among 25,257 blood donors was 30%. Most cases of asthma (95%) were diagnosed by a doctor, 3.5% had current asthma, and 25% of the allergic participants reported asthma.

### 3.2 Association between birth year and AR, AC, and asthma

The prevalence of AR, AC, and asthma among participants younger than 45 years was 20.9% (95% CI: 20.5%–21.4%), 16.8% (95% CI: 16.4%–17.2%), and 11.2% (95% CI: 10.9%–11.6%), respectively.

Comparing participants above 45 years with participants younger than 45 years, the latter had the highest proportion of allergic sensitization (33% vs. 24%), parental allergy (23% vs. 8%), current smokers (14% vs. 12%), females (50% vs. 46%), and current large municipality residence (49% vs. 24%). However, they had a lower proportion of participants growing up on farms with livestock (12% vs. 19%).

**FIGURE 1:**
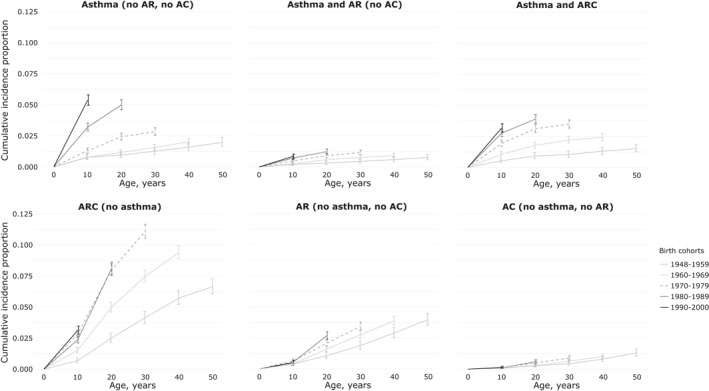
Cumulative incidence proportions in single diseases and multimorbidity. Cumulative incidence proportions (and corresponding 95% confidence intervals) of self‐reported asthma, allergic rhinitis (AR), and allergic conjunctivitis (AC) by age in 10‐year birth cohorts in the overall study population, irrespective of sex. Stratified in distinct single diseases and multimorbidity groups. In multimorbidity, groups age refers to the age of first symptom irrespective of what disease. ARC, both AR and AC

**FIGURE A2:**
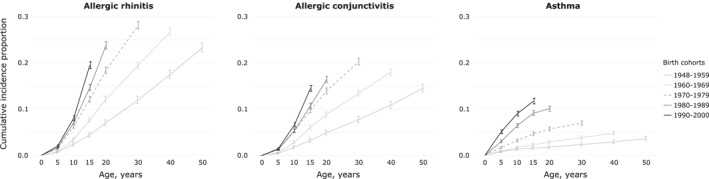
Cumulative incidence proportions in each of the birth cohorts. Cumulative incidence proportions (and corresponding 95% confidence intervals) of self‐reported asthma, allergic rhinitis (AR), and allergic conjunctivitis (AC) by age in ten‐year birth cohorts in the overall study population, irrespective of sex. Participants can appear in more than one symptom group

The cumulative incidence proportions of single diseases and multimorbidity were highest in the youngest birth cohorts, see Figure 1. We were not able to illustrate the cumulative incidence proportions for “asthma and AC” due to the low number of participants in this group. The cumulative incidence proportions of symptoms of AR, AC, and asthma regardless of multimorbidity were also highest in the youngest birth cohorts, se Figure A2. Stratifying by the total number of blood donations had no major influence on the effects of the birth year (Table S1), and the total number of blood donations was included as a confounder further on, see Tables 2 and A1. The youngest birth cohorts had the highest risk of experiencing “only asthma,” “asthma and ARC,” and “ARC,” see Table 2. No associations between birth year and debut of symptoms were seen for those with either AR or AC. The youngest birth cohorts had also the highest risk of experiencing symptoms of asthma, AR, and AC regardless of multimorbidity and they experienced asthma symptoms at a significantly earlier age (*p* = 2.48 × 10^−26^), see Figures A2 and S1.

**TABLE 2:** Association between birth cohorts and the age at the time of first symptom of asthma, AR or AC in single diseases and multimorbidity

		Cohort effect	Symptom age 0–5	Symptom age 6–10	Symptom age 11–15	Symptom age 16–20	Symptom age 21–30	Symptom age 31–40	Symptom age 41–50	Symptom age >50
Asthma (no AR, no AC)										
Birth cohorts	*N*	HR (95% CI)	Reference	HR (95% CI)	HR (95% CI)	HR (95% CI)	HR (95% CI)	HR (95% CI)	HR (95% CI)	HR (95% CI)
1948–1959	6038	1	1	1	1	1	1	1	1	1
1960–1969	10,670	0.87 (0.56–1.37)	1	1.23 (0.60–2.54)	2.42 (0.82–7.14)	2.49 (0.91–6.80)	1.57 (0.76–3.24)	1.52 (0.76–3.03)	1.40 (0.70–2.79)	1.04 (0.44–2.46)
1970–1979	10,648	1.46 (0.97–2.21)	1	1.11 (0.57–2.18)	5.94 (2.20–16.0)	2.86 (1.11–7.38)	1.03 (0.51–2.07)	1.00 (0.51–1.96)	1.50 (0.69–3.28)	‐
1980–1989	9881	**2.80 (1.89–4.14)**	1	1.89 (1.01–3.51)	5.03 (1.90–13.3)	1.78 (0.70–4.54)	0.61 (0.30–1.21)	‐	‐	‐
1990–2000	10,008	**5.71 (3.90–8.36)**	1	1.12 (0.61–2.05)	2.87 (1.09–7.53)	0.85 (0.33–2.15)	0.21 (0.08–0.53)	‐	‐	‐

Asthma and AR (no AC)										
Birth cohorts	*N*	HR (95% CI)	Reference	HR (95% CI)	HR (95% CI)	HR (95% CI)	HR (95% CI)	HR (95% CI)	HR (95% CI)	HR (95% CI)
1948–1959	6074	1	1	1	1	1	1	1	1	1
1960–1969	10,702	0.93 (0.27–3.16)	1	2.00 (0.38–10.5)	4.01 (0.59–27.5)	4.34 (0.64–29.6)	1.27 (0.25–6.45)	0.83 (0.18–3.98)	4.39 (0.39–49.9)	‐
1970–1979	10,659	2.04 (0.68–6.06)	1	1.77 (0.39–8.00)	2.49 (0.41–15.3)	2.04 (0.33–12.7)	1.12 (0.26–4.83)	0.27 (0.06–1.21)	‐	‐
1980–1989	9893	4.10 (1.45–11.6)	1	1.09 (0.25–4.66)	2.30 (0.40–13.3)	0.45 (0.07–2.91)	0.15 (0.03–0.74)	‐	‐	‐
1990–2000	10,025	4.12 (1.45–11.7)	1	0.72 (0.17–3.12)	1.27 (0.22–7.42)	0.46 (0.07–2.91)	‐	‐	‐	‐

Asthma and AC (no AR)										
Birth cohorts	*N*	HR (95% CI)	Reference	HR (95% CI)	HR (95% CI)	HR (95% CI)	HR (95% CI)	HR (95% CI)	HR (95% CI)	HR (95% CI)
1948–1959	6085	1	1	1	1	1	1	1	1	1
1960–1969	10,713	1.61 (0.17–15.5)	1	‐	‐	‐	‐	‐	‐	‐
1970–1979	10,668	2.94 (0.35–26.6)	1	‐	‐	‐	‐	‐	‐	‐
1980–1989	9895	4.78 (0.60–38.1)	1	0.60 (0.05–7.65)	‐	‐	‐	‐	‐	‐
1990–2000	10,033	4.72 (0.58–38.5)	1	0.60 (0.05–7.65)	0.71 (0.04–13.4)	‐	‐	‐	‐	‐

Asthma and ARC										
Birth cohorts	*N*	HR (95% CI)	Reference	HR (95% CI)	HR (95% CI)	HR (95% CI)	HR (95% CI)	HR (95% CI)	HR (95% CI)	HR (95% CI)
1948–1959	6048	1	1	1	1	1	1	1	1	1
1960–1969	10,684	3.47 (1.35–8.92)	1	0.63 (0.23–1.75)	0.63 (0.23–1.70)	0.46 (0.17–1.24)	0.43 (0.16–1.13)	0.39 (0.15–1.04)	0.39 (0.14–1.07)	0.19 (0.05–0.67)
1970–1979	10,640	**6.32 (2.54–15.7)**	1	0.59 (0.22–1.57)	0.46 (0.18–1.19)	0.40 (0.16–1.05)	0.29 (0.11–0.73)	0.21 (0.08–0.54)	0.18 (0.06–0.52)	‐
1980–1989	9881	4.95 (1.97–12.4)	1	0.58 (0.22–1.57)	0.57 (0.22–1.50)	0.54 (0.21–1.42)	0.32 (0.12–0.81)	0.29 (0.11–0.79)	‐	‐
1990–2000	10,017	**5.63 (2.26–14.0)**	1	0.57 (0.21–1.51)	0.64 (0.25–1.65)	0.42 (0.16–1.08)	0.20 (0.08–0.54)	‐	‐	‐

ARC (no asthma)										
Birth cohorts	*N*	HR (95% CI)	Reference	HR (95% CI)	HR (95% CI)	HR (95% CI)	HR (95% CI)	HR (95% CI)	HR (95% CI)	HR (95% CI)
1948–1959	6081	1	1	1	1	1	1	1	1	1
1960–1969	10,702	1.62 (0.89–2.96)	1	1.45 (0.63–3.34)	1.34 (0.54–3.35)	0.78 (0.32–1.93)	2.53 (0.83–7.67)	0.54 (0.21–1.38)	0.71 (0.16–3.22)	‐
1970–1979	10,652	**3.03 (1.72–5.33)**	1	1.30 (0.59–2.86)	1.15 (0.49–2.75)	0.59 (0.25–1.38)	1.01 (0.34–3.03)	**0.13 (0.04–0.35)**	‐	‐
1980–1989	9888	**3.38 (1.93–5.92)**	1	1.26 (0.58–2.76)	0.98 (0.42–2.32)	0.29 (0.12–0.69)	0.44 (0.14–1.38)	‐	‐	‐
1990–2000	10,016	**3.21 (1.83–5.63)**	1	1.38 (0.63–2.99)	0.92 (0.39–2.16)	**0.16 (0.06–0.39)**	‐	‐	‐	‐

AR (no asthma, no AC)										
Birth cohorts	*N*	HR (95% CI)	Reference	HR (95% CI)	HR (95% CI)	HR (95% CI)	HR (95% CI)	HR (95% CI)	HR (95% CI)	HR (95% CI)
1948–1959	5981	1	1	1	1	1	1	1	1	1
1960–1969	10,633	0.57 (0.14–2.26)	1	2.12 (0.47–9.50)	3.82 (0.86–17.0)	3.27 (0.75–14.2)	2.45 (0.59–10.2)	2.06 (0.50–8.55)	1.84 (0.44–7.66)	3.41 (0.77–15.1)
1970–1979	10,629	1.24 (0.38–4.02)	1	1.55 (0.42–5.64)	2.10 (0.57–7.67)	1.46 (0.41–5.25)	1.35 (0.40–4.59)	1.30 (0.38–4.40)	0.88 (0.24–3.21)	‐
1980–1989	9870	1.40 (0.44–4.47)	1	0.94 (0.26–3.43)	2.22 (0.62–7.94)	2.43 (0.70–8.46)	1.51 (0.45–5.02)	0.73 (0.20–2.67)	‐	‐
1990–2000	10,014	0.91 (0.27–3.12)	1	1.31 (0.34–5.08)	4.29 (1.13–16.3)	3.64 (0.98–13.5)	2.31 (0.63–8.43)	‐	‐	‐

AC (no asthma, no AR)										
Birth cohorts	*N*	HR (95% CI)	Reference	HR (95% CI)	HR (95% CI)	HR (95% CI)	HR (95% CI)	HR (95% CI)	HR (95% CI)	HR (95% CI)
1948–1959	6110	1	1	1	1	1	1	1	1	1
1960–1969	10,645	1.14 (0.10–12.5)	1	‐	3.25 (0.19–54.7)	0.91 (0.07–12.3)	2.12 (0.17–26.3)	1.19 (0.10–14.3)	0.77 (0.07–9.01)	0.81 (0.07–10.0)
1970–1979	10,640	0.56 (0.03–8.87)	1	1.80 (0.09–35.3)	9.99 (0.44–228)	3.15 (0.17–58.2)	4.46 (0.25–78.9)	4.16 (0.24–70.8)	1.80 (0.10–32.1)	‐
1980–1989	9875	1.16 (0.10–12.8)	1	0.80 (0.06–11.3)	4.24 (0.26–70.6)	1.98 (0.15–25.7)	3.04 (0.25–37.6)	2.35 (0.19–29.8)	‐	‐
1990–2000	10,016	2.02 (0.22–18.2)	1	0.50 (0.04–5.73)	4.00 (0.29–54.6)	1.14 (0.11–12.1)	2.18 (0.21–22.8)	‐	‐	‐

*Notes:* Poisson proportional hazard regression. Adjusted for number of blood donations (1–10, 11–20, >20), sex, smoking behavior (current, former or never smoker), parental allergy (yes/no), and BMI (continuous). Results are presented as hazard ratios (HR) with corresponding 95% CI. The oldest birth cohort (1948–1959) and symptom age 0–5 years were references. There were no interactions and overall pooled estimates of the effect were calculated. Significant differences after Bonferroni correction are shown in bold. Participants can only appear in one phenotype group. For phenotype groups with multimorbidity, symptom age = age at the time of first symptom of what came first of either asthma, AR or AC.

Abbreviations: AC, allergic conjunctivitis; AR, allergic rhinitis; ARC, both AR and AC; BMI, body mass index; CI, confidence intervals; HR, hazard ratio.

### 3.3 Association between inhalant allergen sensitization and AR, AC, and asthma

**FIGURE A3:**
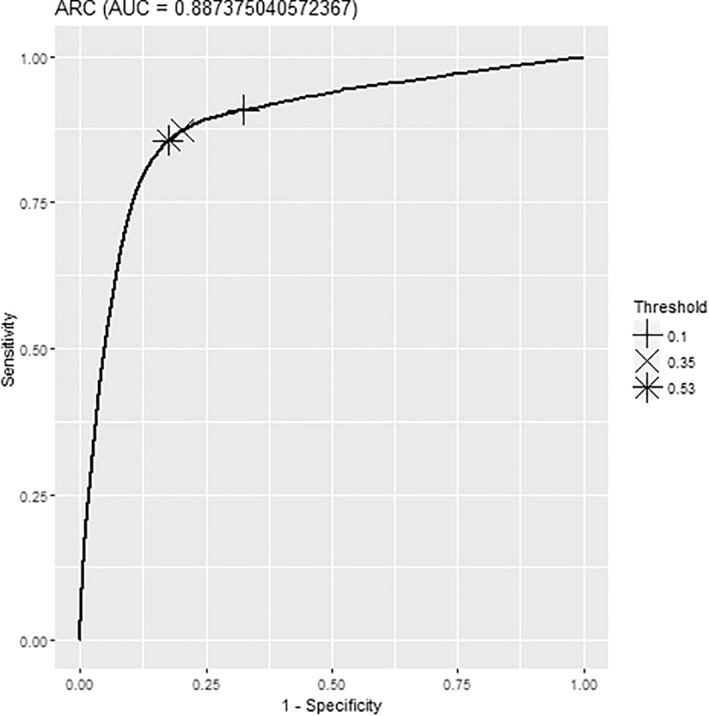
ROC curve analysis. Association between different Phadiatop cutoff values and self‐reported allergic rhinitis and allergic conjunctivitis (ARC) analyzed by receiver operating characteristic (ROC) curve with reported area under the curve (AUC)

The overall seroprevalence (inhalant allergen sensitization) was 30% (34% in males and 25% in females). Participants with only AR had a seroprevalence similar to those with only AC (female: 48% vs. 49%; male: 67% vs. 70%), but the seroprevalence in participants with ARC was substantially higher (female: 80%; male: 91%), see Table A2. Participants with ARC and asthma had the highest seroprevalence (female: 86%; male: 95%), and the combined sensitivity was 91%. Among participants without AR, AC, asthma, nasal‐, eye‐, and respiratory symptoms 14% were sensitized (specificity 86%). Changing the cut‐off value of a positive Phadiatop result^31^ did not improve the overall performance of the test, see ROC curve analysis in Figure A3.

### 3.4 Risk factors for AR, AC, and asthma

Differences between phenotype groups were present for inhalant allergen sensitization, allergic predisposition, current age, sex, smoking, and geographical residence, see Tables A2 and A3.

The multinomial logistic regression analysis confirmed that the inhalant allergen sensitized participants had a higher risk of having asthma, AR, AC and ARC than non‐sensitized participants as compared to the controls, while adjusting for parental allergy, current municipality residence, obesity, smoking behavior, number of blood donations, and age (RRR range: 1.8–36.2 in females and 2.9–82.2 in males), see Table 3. Parental allergy also increased the risk of all phenotypes (RRR range: 1.7–3.8). Age younger than 45 increased the risk of non‐allergic asthma, and obesity showed a trend of increased risk of asthma. Conversely, current smoking had a protective effect on ARC in male participants, see Tables 3, S2a and S2b.

**TABLE 3:** Risk factors for allergic rhino‐conjunctivitis and asthma by multinomial logistic regression analysis

	Asthma (no AR, no AC)	Asthma, and either AR or AC	Asthma and ARC	ARC (no asthma)	Either AR or AC (no asthma)
	RRR (95% CI)	RRR (95% CI)	RRR (95% CI)	RRR (95% CI)	RRR (95% CI)
Male (*N* = 13,299)					
Non‐sensitized	1	1	1	1	1
Sensitized	**2.86 (2.38–3.44)**	**13.8 (9.39–20.4)**	**82.2 (52.2–129)**	**42.3 (35.0–51.0)**	**8.58 (7.27–10.1)**
No parental allergy	1	1	1	1	1
Parental allergy	**1.67 (1.32–2.11)**	**2.69 (1.82–3.96)**	**3.78 (2.98–4.78)**	**2.77 (2.35–3.26)**	**2.15 (1.74–2.64)**
Small municipality	1	1	1	1	1
Large municipality	1.12 (0.93–1.35)	0.96 (0.68–1.35)	0.88 (0.71–1.09)	0.98 (0.85–1.13)	1.08 (0.91–1.27)
Non obese	1	1	1	1	1
Obese	1.47 (1.15–1.88)	1.33 (0.83–2.12)	1.30 (0.96–1.77)	0.86 (0.69–1.06)	1.14 (0.90–1.45)
Never smoker	1	1	1	1	1
Current smoker	1.09 (0.85–1.41)	1.05 (0.66–1.66)	**0.54 (0.38–0.78)**	**0.63 (0.51–0.78)**	0.71 (0.55–0.92)
Former smoker	1.08 (0.86–1.35)	0.92 (0.60–1.42)	0.88 (0.67–1.15)	0.86 (0.72–1.01)	0.96 (0.79–1.17)
>10 blood donations	1	1	1	1	1
1–10 blood donations	**1.46 (1.17–1.82)**	1.17 (0.77–1.78)	1.33 (1.02–1.73)	0.99 (0.83–1.19)	0.93 (0.74–1.18)
45 years or older	1	1	1	1	1
Age below 45 years	**1.72 (1.38–2.14)**	1.40 (0.94–2.08)	1.19 (0.93–1.53)	0.93 (0.80–1.08)	0.79 (0.66–0.95)
Female (*N* = 11,958)					
Non‐sensitized	1	1	1	1	1
Sensitized	**1.82 (1.47–2.27)**	**9.39 (6.69–13.2)**	**36.2 (26.5–49.4)**	**26.1 (22.3–30.6)**	**6.21 (5.23–7.38)**
No parental allergy	1	1	1	1	1
Parental allergy	**1.64 (1.32–2.03)**	**2.00 (1.36–2.92)**	**3.28 (2.55–4.23)**	**2.65 (2.25–3.12)**	**2.31 (1.89–2.82)**
Small municipality	1	1	1	1	1
Large municipality	0.86 (0.72–1.04)	0.91 (0.64–1.29)	1.12 (0.88–1.42)	0.93 (0.80–1.07)	0.84 (0.70–1.01)
Non‐obese	1	1	1	1	1
Obese	1.41 (1.11–1.79)	**2.36 (1.59–3.48)**	1.35 (0.97–1.88)	1.03 (0.84–1.27)	1.05 (0.83–1.34)
Never smoker	1	1	1	1	1
Current smoker	1.37 (1.08–1.73)	1.05 (0.64–1.70)	1.10 (0.79–1.54)	0.88 (0.71–1.09)	1.12 (0.88–1.43)
Former smoker	1.02 (0.80–1.29)	1.27 (0.85–1.90)	1.24 (0.93–1.65)	1.09 (0.91–1.29)	1.11 (0.91–1.36)
>10 blood donations	1	1	1	1	1
1–10 blood donations	**1.50 (1.23–1.82)**	1.29 (0.88–1.88)	1.15 (0.88–1.49)	1.03 (0.87–1.22)	0.78 (0.63–0.97)
45 years or older	1	1	1	1	1
Age below 45 years	**2.08 (1.64–2.63)**	1.46 (0.97–2.20)	1.26 (0.94–1.68)	0.94 (0.80–1.11)	0.87 (0.72–1.06)

*Notes:* Results are presented as adjusted relative risk ratios (RRR) with 95% CI. One analysis for each sex adjusted for all covariates was performed. Controls served as base. Significant results after Bonferroni correction are shown in bold. Sensitized: sIgE (Phadiatop) ≥ 0.35 kU/L. Non‐sensitized: sIgE (Phadiatop) < 0.35 kU/L.

Abbreviations: AC, allergic conjunctivitis; AR, allergic rhinitis; ARC, both AR and AC; CI, confidence intervals; sIgE, allergen‐specific immunoglobulin E.

### 3.5 Associations between geographical upbringing and AR, AC, asthma, and allergen sensitization

Participants who grew up on farms with livestock had the lowest proportions of AR, AC, and inhalant allergen sensitization compared with the other urbanization categories, see Table A3 and A4 for characteristics of the study population stratified by phenotype group and inhalant allergen sensitization, respectively. Participants who grew up in inner cities had a higher proportion of parental allergy compared with those from farms with livestock (27% vs. 16%), and most of the participants from inner cities were residents in a large density populated municipality.

**FIGURE 2:**
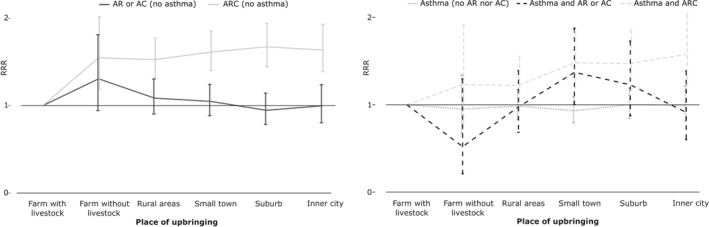
Risk of allergic rhinitis (AR), allergic conjunctivitis (AC), and asthma in relation to geographical upbringing. Multinomial logistic regression analysis adjusted for sex, age, smoking behavior, parental allergy, body mass index, total number of blood donations, and current municipality residence in participants younger than 45 years (*N* = 32,218). Results are presented as relative risk ratio with corresponding 95% confidence interval

**FIGURE 3:**
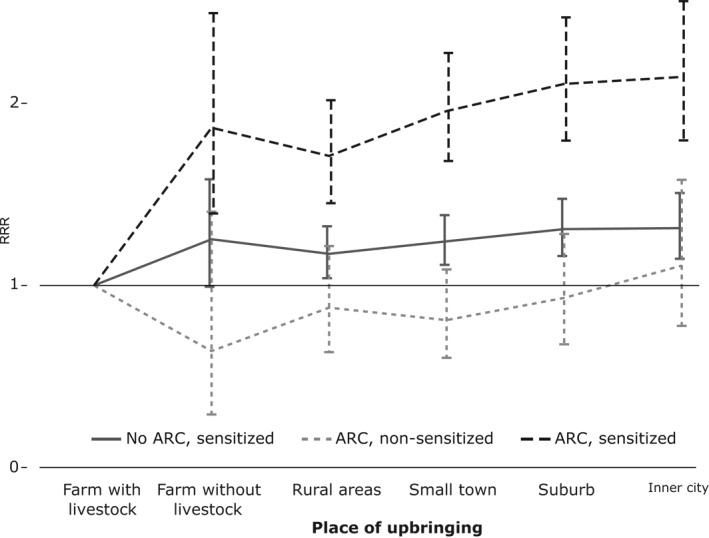
Risk of ARC and IgE sensitization in relation to geographical upbringing. Multinomial logistic regression analysis adjusted for sex, age, smoking behavior, parental allergy, body mass index, total number of blood donations, and current municipality residence in participants with Phadiatop measurements (*N* = 25,257). Results are presented as relative risk ratios with corresponding 95% confidence interval. ARC, both allergic rhinitis and allergic conjunctivitis

The multinomial logistic regression analysis among participants younger than 45 years showed that the protective effect of living on a farm with livestock during childhood was only present for acquiring ARC (with or without asthma) compared with almost all other urbanization categories, see Figure 2 and Table S3. No effect on non‐allergic asthma by having either AR or AC was demonstrated. Based on that, ARC and inhalant allergen sensitization were further analyzed regardless of asthma and without age stratification. Farm childhood only had a protective effect on ARC in inhalant allergen sensitized participants, see Figure 3 and Table S4.

### 3.6 Risk factors for inhalant allergen sensitization

The seroprevalence (inhalant allergen sensitization) was positively associated with male sex, allergic predisposition, age younger than 45 years, and living in a large density populated municipality, see Table A4. The seroprevalence in adulthood increased with decreasing age at first symptoms of AR and AC in participants with ARC.

The multivariable logistic regression analysis confirmed that males had a higher risk of testing seropositive than females (OR = 1.57, 95% CI: 1.49–1.67), while adjusting for parental allergy, current municipality residence, obesity, smoking behavior, number of blood donations, and current age, see Table 4. The analysis also showed independent effect of parental allergy, age, smoking, and current municipality residence. The risk of testing seropositive was higher in participants with parental allergy compared with those without (OR = 2.22, 95% CI: 2.08–2.39), in participants younger than 45 years compared with participants 45 years or older (OR = 1.33, 95% CI: 1.25–1.42), and in participants currently living in large compared with small density populated municipalities (OR = 1.12, 95% CI: 1.06–1.19). However, current and former smoking had a protective effect compared with never smoking (OR = 0.87, 95% CI: 0.80–0.95; OR = 0.85, 95% CI: 0.79–0.91, respectively).

**TABLE 4:** Multivariable logistic regression analysis of risk factors of inhalant allergen sensitization (*N* = 25,257)

	Sensitized	Sensitized
	Crude OR (95% CI)	Adjusted OR (95% CI)
Female (reference)	1	1
Male	**1.53 (1.45–1.61)**	**1.57 (1.49–1.67)**
No parental allergy (reference)	1	1
Parental allergy	**2.34 (2.19–2.51)**	**2.22 (2.08–2.39)**
Small municipality (reference)	1	1
Large municipality	**1.27 (1.21–1.35)**	**1.12 (1.06–1.19)**
Non obese (reference)	1	1
Obesity	0.89 (0.82–0.97)	0.95 (0.87–1.03)
Never smoker (reference)	1	1
Current smoker	**0.86 (0.79–0.93)**	**0.87 (0.80–0.95)**
Former smoker	**0.77 (0.72–0.82)**	**0.85 (0.79–0.91)**
>10 blood donations (reference)	1	1
1–10 blood donations	**1.16 (1.08–1.24)**	0.99 (0.92–1.06)
45 years or older (reference)	1	1
Age below 45 years	**1.54 (1.45–1.63)**	**1.33 (1.25–1.42)**

*Note:* Results are presented as OR with 95% CI. Crude: univariate logistic regression without adjustment. Adjusted: adjustment for sex, parental allergy, municipality, obesity, smoking behavior, total number of blood donations, and age. Significant results after Bonferroni correction are shown in bold. Sensitized: sIgE (Phadiatop) ≥ 0.35 kU/L.

Abbreviations: CI, confidence intervals; OR, odds ratios; sIgE, allergen‐specific immunoglobulin E.

### 3.7 Seasonal allergy and asthma

**FIGURE A4:**
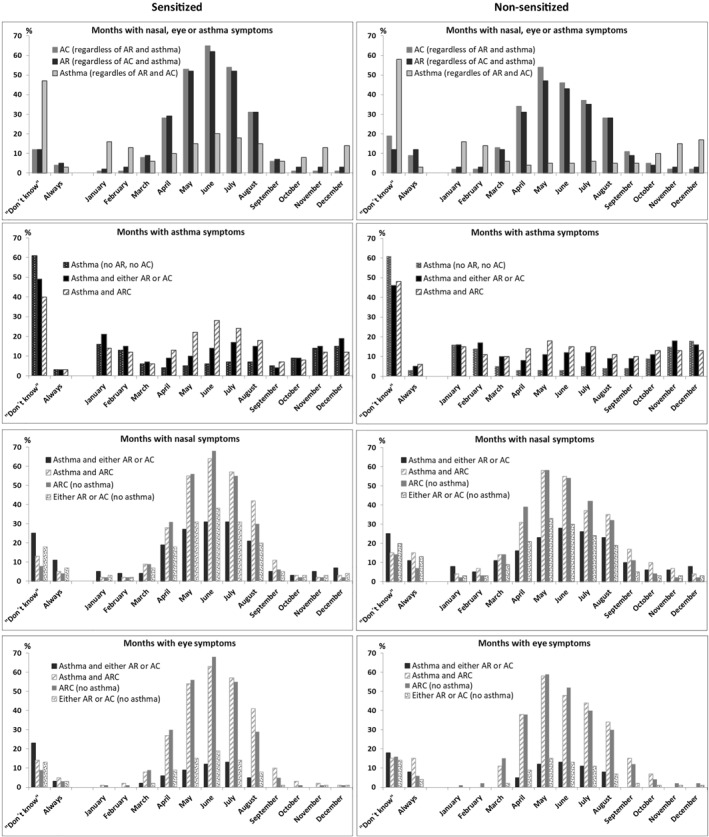
Months with symptoms of allergy and/or asthma. Nasal‐, eye‐ or asthma symptoms in participants with allergic rhinitis (AR), allergic conjunctivitis (AC), and asthma (*n* = 6278) stratified by inhalant allergen sensitization. Results are presented as proportions of the allergic and/or asthmatic participants. The figure shows both sexes in one. Participants could give more than one answer and appear in more than one symptom group

Most allergic participants had intermittent symptoms, and the months during which they usually experienced allergic symptoms were from April to August (corresponding to the hay fever season) with the highest proportion in June (grass pollen season in Denmark), see Figure A4. The proportions of participants with symptoms were generally higher in sensitized compared with non‐sensitized participants. Persistent AR and AC symptoms were more frequent in participants with either AR or AC compared to those with ARC, as well as in sensitized compared with non‐sensitized. Symptoms of both allergic and non‐allergic asthma peaked during the winter; allergic asthma also peaked in the summer.

### 3.8 Missing data

The number of unanswered questions in the questionnaire was approximately 4%.

The study population (*N* = 52,976) comprised 3.4% (*N* = 1780) participants with missing data in at least one of the following variables: asthma, AR, AC, smoking, BMI, parental allergy, and place of upbringing. Of these, 1468 had missing data in one variable, 301 in two to three variables, and 11 in four to seven of the variables. All participants had complete data on age, sex, current municipality residence, and total number of blood donations.

## 4 DISCUSSION

In this cohort of Danish blood donors, the prevalence of AR, AC, asthma, and inhalant allergen sensitization was 19%, 15%, 9%, and 30%, respectively. The prevalence of inhalant allergen sensitization, allergic asthma, non‐allergic asthma, and ARC increased over time and the youngest birth cohorts experienced asthma symptoms at an earlier age. Moreover, inhalant allergen sensitization and allergic predisposition increased the risk of the diseases. Allergic multimorbidity was associated with inhalant allergen sensitization and participants with ARC had a higher sIgE seroprevalence than participants with either AR or AC, indicating that self‐reported ARC represent a primarily sIgE‐positive phenotype, while those with either AR or AC represent a more diverse phenotypes. Farm upbringing had a protective effect on acquiring ARC but only in inhalant allergen sensitized participants. Inhalant allergen sensitization was associated with male sex, allergic predisposition, and age younger than 45 years. As expected seasonal AR was frequently accompanied by AC.^32^

Among participants younger than 45 years, the prevalence of AR was 20.9%, 95% CI: 20.5%–21.4%, thus slightly lower than in a previous Danish study (23.5%, 95% CI: 22.5%–24.5%)^1^ and in the RHINE study (24.0%, 95% CI: 23.2%–24.6%)^2^ that are based on the same ECRHS questions in individuals aged 20–44 years. However, the prevalence of asthma of 11.2%, 95% CI: 10.9%–11.6% was similar to the previous Danish study (11.3%, 95% CI: 10.6%–12.0%),^1^ as well as the proportion (95%) reporting that asthma was diagnosed by a doctor.

The lower disease prevalence in participants aged 45 or older may be related to the lower prevalence of the risk factors, for example, inhalant allergen sensitization and allergic predisposition, as well as the healthy donor effect.^33^, ^34^, ^35^ Few studies have examined the age effect on the coexistence of asthma and allergic diseases. Similar to other studies, our results showed increased risk of experiencing asthma, AR, or AC symptoms, in the youngest compared with the oldest birth cohort for participants with ARC and allergic asthma. However, contrary to other studies, this was also seen for asthma only.^36^ In this study, we assessed both temporal and age‐related changes. Changes in symptoms and diseases may be related to changes in life style,^37^ microbial exposure in the environment,^38^ and changes in healthcare patterns, for example, changes in perception of illness and diagnostic practices over time.

A diverse and mature microbiota lowers the prevalence of atopy.^39^, ^40^, ^41^, ^42^ Hence, the urbanization categories were used as proxy measures for microbial load and diversity early in life.^1^ Consistent with previous studies, growing up on farms (an environment rich in microbial load) was associated with lower risk of allergic sensitization, AR, and allergic asthma in adulthood.^2^, ^10^ Asthma, AR, and AC often coexist in the same individuals (multimorbidity). When stratifying our study population into two allergic groups, we found that farm childhood only had a protective effect on acquiring ARC (allergic multimorbidity) with and without asthma and not on acquiring either AR or AC. Combining self‐reported data with data on inhalant allergen sensitization showed that the effect was only present in sensitized participants with ARC. We speculate that the protective effect of farm childhood on allergic disease in adulthood is explained by the protective effect on allergic sensitization which strongly correlates with ARC.

We further speculate that the parental allergy factor may also impact the results. A Swedish study showed that men with allergy more often chose to move from farms compared to those without allergy, resulting in a healthy worker effect with less atopy and less severe symptoms in the farming population.^43^ In our study population, parental allergy was less frequent in participants from farms compared to participants from inner city and most of the participants from inner cities were currently residents in a large municipality (with higher exposure to air pollution). Nevertheless, adjusting for parental allergy and current municipality residence only slightly altered the results. Use of self‐reported exposures and outcomes, in particular dating back to early life, may induce recall bias. However, we expect that participants were able to remember place of upbringing with a reasonable accuracy.

An association between obesity and asthma, especially, in females may exist. Though, the causality between obesity and asthma is still uncertain; a suggested explanation is due to changes in systemic metabolism.^15^, ^44^, ^45^

The overall seroprevalence of inhalant allergen sensitization was 30% which is consistent with other studies.^9^, ^10^ As expected, inhalant allergen sensitization was positively associated with male sex, allergic predisposition, and younger age,^11^, ^46^, ^47^ while current and former smoking had a protective effect.^48^ The overall sensitivity of the Phadiatop analysis was 91% among participants with both ARC and asthma. This is in accordance with other studies showing that polysensitization is higher in individuals with allergic multimorbidity than in those with single diseases.^49^, ^50^, ^51^ The Phadiatop analysis includes allergens from the nine most common respiratory sensitizers, but the participants could be sensitized to other allergens, have local AR,^52^ or they could represent a non‐allergic phenotype.

We found a strong association between allergic multimorbidity and asthma. It is, however, a limitation of our study that severe asthmatic individuals are not included. Allergic multimorbidity is tightly associated with severe asthma.^53^, ^54^, ^55^, ^56^ The absence of participants with severe asthma in our study population could cause underestimation of the prevalence of asthma with allergic multimorbidity (AR and AC) and explain why only 47% of our asthmatic participants have concomitant AR, while this proportion is 64.5% in another study using the ECRHS cohort.^57^

The strength of our study is a large, overall healthy, and well‐characterized study population. We included a detailed standardized questionnaire and an objective marker of allergic sensitization. The DBDS relies on the existing infrastructure of continuous collection of blood samples in the Danish blood centers and on the local staff at the blood centers, who collect questionnaire data during blood donation. The participation rate was high,^24^ and the number of unanswered questions in the questionnaire is low (approximately 3%). Prior to study inclusion, the questions were unknown to the blood donors. All participants in our study were unremunerated voluntary blood donors and had no incentive to non‐disclosure of diseases. Although the cross‐sectional design hinders causal inference, it allows exploration of associations to generate hypotheses for further research.

## 5 CONCLUSION

Birth year, childhood residence, inhalant allergen sensitization, and allergic predisposition had an effect on the risk of asthma, AR, and AC. We found that individuals with self‐reported ARC represent a primarily sIgE phenotype while those with either AR or AC represent more diverse phenotypes, and we suggest that the two phenotypes are analyzed separately in epidemiological studies based on only self‐reported allergy.

The DBDS blood donor population proves well‐suited for further studies of gene–environment interactions and biomarkers related to airborne allergy and asthma.

## CONFLICT OF INTEREST

Henrik Ullum has received unrestricted departmental grants from Novartis. No other conflicts of interest declared. None of the funders had any influence on study design, data collection and analysis, decision to publish, or preparation of this manuscript.

## AUTHOR CONTRIBUTIONS

Susan Mikkelsen, Christian Erikstrup and Torben Sigsgaard designed the study. Susan Mikkelsen drafted the manuscript and performed the statistical analysis with contribution from Jens Kjærgaard Boldsen, Christian Erikstrup, Torben Sigsgaard, Gitte Juel Holst, Henrik Hjalgrim, Klaus Rostgaard , and Khoa Manh Dinh. All authors interpreted and discussed the results. Christian Erikstrup, Henrik Ullum, Ole Birger Pedersen, Kristoffer Sølvsten Burgdorf , Thomas Folkmann Hansen , Kaspar Rene Nielsen, Helene Martina Paarup , Mikkel Steen Petersen , Erik Sørensen , Karina Banasik , Kathrine Agergård Kaspersen, Khoa Manh Dinh, Bjarne Kuno Møller , Linda Jenny Handgaard and Susan Mikkelsen made substantial contribution to acquisition of data. All authors contributed to revising the manuscript critically and approved the final draft for publication.

## Supporting information

Supplementary Material S1

Supplementary Material S2

## 1 Questionnaire

### 1.1

**Table:**

**Questions**	**Definition**
“Do you have any nasal allergies including hay fever?”	Allergic rhinitis (AR)
In addition an affirmative answer to:	
“In which months of the year do this nose problem occur?”	Months with nasal symptoms
“How old were you when you first had hay fever or nasal allergy?”	First nasal symptoms
“When were the last time you had nasal symptoms?”	Last nasal symptoms
“Have you ever had a problem with sneezing, or a runny or a blocked nose when you did not have a cold or the flu?”	Nasal symptoms
“Do you have any eye allergies including hay fever?”	Allergic conjunctivitis (AC)
In addition an affirmative answer to:	
“In which months of the year do this eye problem occur?”	Months with eye symptoms
“How old were you when you first had hay fever or eye allergy?”	First eye symptoms
“When were the last time you had eye symptoms?”	Last eye symptoms
“Have you ever had a problem with itchy or watery eyes?”	Eye symptoms
“Do you have any nasal allergies including hay fever?” AND “Do you have any eye allergies including hay fever?”	Allergic rhino‐conjunctivitis (ARC)
“Do you have or have you ever had asthma?”	Asthma
In addition an affirmative answer to:	
“Have you ever had asthma diagnosed by a doctor?”	Doctor‐diagnosed asthma
“Which months of the year do you usually have trouble with your breathing?”	Months with asthma symptoms
“How old were you when you had your first attack of asthma?”	First asthma symptom
At least one of the following:“Are you currently taking any medicine (including inhalers, aerosols or tablets) for asthma?”“Have you been woken by an attack of coughing at any time in the last 12 months?”“Have you been woken by an attack of shortness of breath at any time in the last 12 months?”“When were the last time you had an attack of asthma?” – With the answer “Within the last 12 month”.	Current asthma
“Have you had wheezing or whistling in your chest at any time?” and “Have you been at all breathless when the wheezing noise was present?”	Respiratory symptoms
No AR, no AC, and no asthma	Controls
“Have one or both of you parents allergy?”	Allergic predisposition
“Which category best describes the place you lived most of the time when you were under the age of five years?”1) a farm with livestock, 2) a farm without livestock, 3) a village in a rural area, 4) a small town, 5) a suburb of city, or 6) inner city of Copenhagen, Aarhus, Odense, Aalborg, or Esbjerg*	Place of upbringing
Current smoking: “Do you smoke?”. Former smoking: “Have you smoked earlier?”. Never smoking: negative answers to the two questions. Smokers stated the duration of smoking (years) and the year of smoking cessation.	Smoking behavior
“How much do you weigh?”	Weight
“How tall are you?”	Height

Note: Body mass index (BMI) (kg/m^2^) was calculated from self‐reported weight and height and obesity was defined as a BMI ≥ 30 kg/m^2^.

^*^The five largest cities in Denmark with 72,000 to 727,000 citizens.

**TABLE A1:** Association between birth cohorts and the age at the time of first symptom of asthma, allergic rhinitis or allergic conjunctivitis (*N* = 52,976)

		**Cohort effect**	**Symptom age 0–5**	**Symptom age 6–10**	**Symptom age 11–15**	**Symptom age 16–20**	**Symptom age 21–30**	**Symptom age 31–40**	**Symptom age 41–50**	**Symptom age > 50**
**a.** **Age at the first** ***symptom of asthma***										
Birth cohorts	*N*	HR (95% CI)	Reference	HR (95% CI)	HR (95% CI)	HR (95% CI)	HR (95% CI)	HR (95% CI)	HR (95% CI)	HR (95% CI)
1948–1959	6256	1	1	1	1	1	1	1	1	1
1960–1969	11,342	1.01 (0.73–1.43)	1	1.52 (0.90–2.57)	2.07 (1.11–4.03)	2.47 (1.30–4.97)	1.53 (0.93–2.53)	1.58 (0.95–2.63)	1.35 (0.82–2.23)	0.99 (0.50–1.87)
1970–1979	11,651	**1.83 (1.36–2.53)**	1	1.38 (0.86–2.25)	**2.81 (1.58–5.26)**	2.31 (1.26–4.49)	1.03 (0.65–1.66)	0.91 (0.56–1.49)	0.82 (0.45–1.48)	‐
1980–1989	11,323	**2.89 (2.17–3.93)**	1	1.84 (1.17–2.94)	**3.02 (1.72–5.57)**	1.26 (0.69–2.45)	0.48 (0.30–0.78)	0.51 (0.26–0.95)	‐	‐
1990–2000	11,074	**4.59 (3.46–6.23)**	1	1.24 (0.79–1.97)	1.85 (1.06–3.39)	0.80 (0.44–1.55)	**0.31 (0.18–0.55)**	‐	‐	‐

**b.** **Age at the first** ***nasal symptom*** ***(allergic rhinitis)***										
Birth cohorts	*N*	HR (95% CI)	Reference	HR (95% CI)	HR (95% CI)	HR (95% CI)	HR (95% CI)	HR (95% CI)	HR (95% CI)	HR (95% CI)
1948–1959	6195	1	1	1	1	1	1	1	1	1
1960–1969	11,316	1.08 (0.75–1.57)	1	1.47 (0.95–2.25)	1.92 (1.26–2.89)	1.60 (1.06–2.39)	1.41 (0.95–2.07)	1.33 (0.90–1.95)	1.54 (1.04–2.25)	1.20 (0.79–1.80)
1970–1979	11,634	**1.82 (1.31–2.58)**	1	1.50 (1.00–2.21)	1.55 (1.05–2.27)	1.31 (0.89–1.90)	1.18 (0.81–1.68)	1.19 (0.83–1.69)	1.16 (0.79–1.68)	‐
1980–1989	11,305	**1.97 (1.43–2.79)**	1	1.40 (0.94–2.06)	1.71 (1.16–2.49)	1.69 (1.15–2.42)	1.73 (1.20–2.44)	1.35 (0.92–1.95)	‐	‐
1990–2000	11,078	**2.22 (1.62–3.13)**	1	1.28 (0.86–1.87)	**2.18 (1.49–3.14)**	**1.93 (1.33–2.76)**	**1.80 (1.25–2.55)**	‐	‐	‐

**c.** **Age at the first** ***eye symptom*** ***(allergic conjunctivitis)***										
Birth cohorts	*N*	HR (95% CI)	Reference	HR (95% CI)	HR (95% CI)	HR (95% CI)	HR (95% CI)	HR (95% CI)	HR (95% CI)	HR (95% CI)
1948–1959	6225	1	1	1	1	1	1	1	1	1
1960–1969	11,323	1.22 (0.78–1.94)	1	1.36 (0.80–2.26)	1.76 (1.05–2.89)	1.36 (0.81–2.23)	1.30 (0.79–2.09)	1.28 (0.78–2.06)	1.24 (0.76–1.99)	1.16 (0.70–1.90)
1970–1979	11,645	**2.58 (1.74–3.98)**	1	1.08 (0.66–1.71)	1.09 (0.67–1.71)	0.95 (0.59–1.49)	0.84 (0.53–1.28)	0.82 (0.52–1.26)	0.91 (0.56–1.42)	‐
1980–1989	11,320	**2.36 (1.59–3.64)**	1	1.06 (0.65–1.68)	1.31 (0.81–2.07)	1.28 (0.80–2.01)	1.28 (0.81–1.95)	1.42 (0.89–2.22)	‐	‐
1990–2000	11,075	**2.34 (1.58–3.61)**	1	1.23 (0.76–1.94)	1.78 (1.10–2.78)	1.66 (1.03–2.59)	1.62 (1.02–2.49)	‐	‐	‐

*Notes*: Poisson proportional hazard regression. Adjusted for number of blood donations (1–10, 11–20, >20), sex, smoking behavior (current, former or never smoker), parental allergy (yes/no), and BMI (continuous). Results are presented as hazard ratios (HR) with corresponding 95% confidence intervals (CI). Symptom age = age at the time of first symptom. The oldest birth cohort (1948–1959) and symptom age 0–5 years were references. There were no interactions and overall pooled estimates of the effect were calculated. Significant differences after Bonferroni correction are shown in bold. Participants can appear in more than one symptom group.

**TABLE A2:** Association between allergic rhino‐conjunctivitis, asthma, and inhalant allergen sensitization (N = 25,257)

Self‐reported diseases and symptoms	Sex	Total	Sensitized
		N	N (%)
AR (no AC and no asthma)	Female	470	231 (49.1)
	Male	593	392 (66.1)
AC (no AR and no asthma)	Female	168	77 (45.8)
	Male	149	105 (70.5)
ARC (no asthma)	Female	1210	972 (80.3)
	Male	1489	1357 (91.1)
Asthma, and either AR or AC	Female	148	89 (60.1)
	Male	154	120 (77.9)
Asthma and ARC	Female	344	294 (85.5)
	Male	441	420 (95.2)
Asthma (no AR, no AC)	Female	524	119 (22.7)
	Male	531	223 (42.0)
No asthma, no AR, and no AC With nasal‐, eye‐, or respiratory symptoms	Female	2571	453 (17.6)
	Male	2650	641 (24.2)
No asthma, no AR, and no AC Without nasal‐, eye‐, and respiratory symptoms	Female	6303	711 (11.3)
	Male	7057	1169 (16.6)
Total^a^	Female	11,958	2994 (25.0)
	Male	13,299	4491 (33.8)

*Note:* Results are presented as numbers and percentages.

Abbreviations: AR, allergic rhinitis; AR, allergic conjunctivitis; ARC: both AR and AC; Sensitized: allergen‐specific immunoglobulin E (Phadiatop) ≥ 0.35 kU/l.

^a^ Total does not sum up the numbers above due to missing data in one of the variables asthma, AR or AC (*N* = 455)

**TABLE A3:** Characteristics of the study population stratified by phenotype groups (*N* = 52,976)

*N* (%)		Controls	Asthma (no AR, no AC)	Asthma, and either AR or AC	Asthma and ARC	ARC (no asthma)	Either AR or AC (no asthma)	Comparison
		38,931 (74.5)	2359 (4.48)	664 (1.26)	1685 (3.19)	5581 (10.6)	2894 (5.53)	

	Sex	Median (IQR)	Median (IQR)	Median (IQR)	Median (IQR)	Median (IQR)	Median (IQR)	
Age	F	39.7 (26.9–51.1)	30.3 (23.8–43.0)	36.2 (26.8–45.8)	33.2 (25.5–44.4)	38.4 (26.6–48.4)	41.8 (28.7–51.4)	**
	M	41.9 (30.3–52.3)	31.6 (25.4–45.0)	34.7 (26.8–45.4)	35.9 (27.0–45.4)	40.2 (30.4–49.1)	42.3 (31.5–51.8)	**
BMI	F	24.3 (22.1–27.5)	24.8 (22.3–28.0)	25.3 (22.6–29.4)	24.6 (22.3–28.2)	24.2 (22.0–27.1)	24.7 (22.3–28.1)	**
	M	25.5 (23.6–28.0)	25.7 (23.7–28.1)	25.5 (23.5–27.8)	25.5 (23.5–28.1)	25.3 (23.4–27.5)	25.5 (23.5–27.8)	*

	Sex	% (95% CI)^a^	% (95% CI)^a^	% (95% CI)^a^	% (95% CI)^a^	% (95% CI)^a^	% (95% CI)^a^	
Females	‐	49.1 (48.6–49.6)	50.6 (48.6–52.6)	47.3 (43.5–51.1)	44.9 (42.6–47.3)	45.7 (44.4–47.0)	48.2 (46.4–50.0)	**
Current smokers	F	13.8 (13.3–14.3)	17.4 (15.4–19.7)	14.7 (11.2–19.0)	15.3 (12.9–18.1)	11.2 (10.0–12.5)	13.4 (11.7–15.3)	**
	M	13.6 (13.1–14.1)	16.3 (14.3–18.5)	15.0 (11.6–19.1)	10.1 (8.28–12.2)	9.51 (8.51–10.6)	11.0 (9.52–12.7)	**
Former smokers	F	23.5 (22.9–24.1)	20.2 (18.0–22.6)	24.5 (20.1–29.6)	21.0 (18.3–24.0)	21.9 (20.4–23.6)	25.3 (23.1–27.7)	NS
	M	22.2 (21.6–22.7)	21.5 (19.3–24.0)	16.3 (12.8–20.5)	18.3 (16.0–20.9)	19.1 (17.7–20.5)	21.3 (19.3–23.4)	**
Large municipality	F	39.2 (38.5–39.9)	41.9 (39.1–44.7)	41.1 (35.8–46.6)	46.6 (43.1–50.2)	40.5 (38.6–42.5)	36.9 (34.4–39.4)	**
	M	37.8 (37.1–38.5)	43.4 (40.6–46.2)	41.4 (36.4–46.7)	42.3 (39.2–45.6)	42.4 (40.7–44.2)	38.8 (36.3–41.3)	**
Obesity	F	14.2 (13.7–14.7)	18.7 (16.6–21.0)	23.4 (19.0–28.4)	17.1 (14.6–20.0)	13.4 (12.1–14.8)	15.7 (13.9–17.7)	**
	M	12.8 (12.4–13.3)	15.5 (13.5–17.7)	11.7 (8.75–15.5)	13.1 (11.0–15.4)	10.9 (9.80–12.0)	12.9 (11.3–14.7)	NS
Parental allergy	F	15.8 (15.2–16.3)	24.9 (22.5–27.4)	33.5 (28.5–38.9)	42.7 (39.2–46.2)	34.4 (32.6–36.2)	25.3 (23.1–27.7)	**
	M	11.6 (11.2–12.1)	19.6 (17.4–22.0)	31.3 (26.7–36.4)	37.8 (34.7–40.9)	28.1 (26.5–29.7)	22.8 (20.7–25.0)	**

	N	% (95% CI)^b^	% (95% CI)^b^	% (95% CI)^b^	% (95% CI)^b^	% (95% CI)^b^	% (95% CI)^b^	
Age strata (years)								
18–24	6531	71.9 (70.9–72.8)	7.85 (7.28–8.45)	1.40 (1.16–1.67)	4.03 (3.62–4.48)	10.6 (9.96–11.3)	4.26 (3.84–4.72)	**
25–34	13,076	72.1 (71.3–72.9)	6.11 (5.70–6.55)	1.70 (1.49–1.95)	4.21 (3.87–4.57)	10.5 (9.98–11.1)	5.37 (4.99–5.79)	
35–44	11,349	72.1 (71.3–73.0)	3.67 (3.34–4.03)	1.49 (1.28–1.73)	3.69 (3.36–4.05)	13.1 (12.5–13.8)	5.88 (5.46–6.33)	
45–54	12,221	76.6 (75.9–77.4)	2.84 (2.55–3.15)	0.95 (0.79–1.14)	2.54 (2.28–2.84)	10.9 (10.3–11.4)	6.17 (5.75–6.62)	
55–67	9799	82.1 (81.3–83.0)	2.51 (2.20–2.87)	0.69 (0.53–0.89)	1.37 (1.14–1.64)	7.53 (6.98–8.12)	5.75 (5.27–6.28)	
Place of upbringing								
Farm with livestock	7764	80.7 (79.8–81.6)	4.42 (3.98–4.91)	0.90 (0.71–1.14)	1.95 (1.66–2.28)	6.72 (6.17–7.30)	5.27 (4.79–5.80)	**
Farm without livestock	1222	76.0 (73.5–78.3)	4.16 (3.17–5.45)	0.67 (0.34–1.31)	2.91 (2.10–4.03)	10.1 (8.50–11.9)	6.16 (4.94–7.67)	
Rural area	10,075	75.4 (74.6–76.3)	4.65 (4.25–5.08)	1.10 (0.91–1.32)	2.90 (2.59–3.25)	10.4 (9.77–11.0)	5.56 (5.12–6.02)	
Small town	17,333	73.5 (72.8–74.1)	4.43 (4.13–4.75)	1.52 (1.34–1.71)	3.57 (3.30–3.86)	11.4 (10.9–11.9)	5.64 (5.30–5.99)	
Suburb	10,372	72.4 (71.5–73.2)	4.67 (4.28–5.10)	1.48 (1.27–1.73)	3.72 (3.37–4.10)	12.4 (11.8–13.1)	5.35 (4.93–5.81)	
Inner city	6006	72.8 (71.7–73.9)	4.66 (4.15–5.23)	1.12 (0.88–1.42)	3.77 (3.31–4.28)	11.7 (10.9–12.6)	5.91 (5.34–6.54)	

*Notes:* Results are presented as percentages with corresponding 95% confidence intervals (CI) and medians with interquartile ranges (IQR). Phenotype groups were compared by Kruskall–Wallis test for non‐normally distributed data and chi‐squared test for categorical data. P‐values were Bonferroni corrected, **p* < 0.05; ***p* < 0.001; NS: non‐significant. Obesity: BMI ≥ 30 kg/m^2^. In total 862 participants could not be categorized in the phenotype groups due to missing data in asthma, AR or AC.

Abbreviations: AR: allergic rhinitis; AC, allergic conjunctivitis; ARC, both AR and AC; BMI, body mass index (kg/m^2^); F, female; M, male.

^a^ Compared with the total number of participants in the column.

^b^ Compared with the total number of participants in the row.

**TABLE A4:** Characteristics of the study population stratified by inhalant allergen sensitization (*N* = 25,257)

*N* (%)		Non‐sensitized	Sensitized	Comparison
		17,772 (70.4)	7485 (29.6)	

	Sex	Median (IQR)	Median (IQR)	
Age	F	40.9 (27.7–51.9)	37.3 (26.3–48.3)	**
	M	43.1 (31.4–53.2)	38.6 (28.8–48.8)	**
BMI	F	24.4 (22.1–27.6)	24.2 (22.0–27.3)	NS
	M	25.5 (23.6–27.8)	25.3 (23.4–27.8)	*

	Sex	% (95% CI)^a^	% (95% CI)^a^	
Females	‐	50.4 (49.7–51.2)	40.0 (38.9–41.1)	**
Current smokers	F	14.6 (13.8–15.3)	13.4 (12.2–14.6)	NS
	M	12.9 (12.3–13.7)	12.4 (11.5–13.5)	NS
Former smokers	F	24.1 (23.2–25.0)	21.0 (19.6–22.5)	*
	M	23.5 (22.6–24.4)	19.1 (17.9–20.2)	**
Large municipality	F	37.9 (36.9–38.9)	41.9 (40.2–43.7)	*
	M	34.8 (33.8–35.8)	42.2 (40.8–43.7)	**
Obesity	F	14.3 (13.6–15.0)	13.2 (12.1–14.5)	NS
	M	12.6 (11.9–13.3)	11.4 (10.5–12.4)	NS
Parental allergy	F	16.1 (15.4–16.9)	29.6 (28.0–31.2)	**
	M	11.3 (10.6–11.9)	23.2 (22.0–24.5)	**
	N	% (95% CI)^b^	% (95% CI)^b^	
Age strata (years)				**
18–24	3493	66.6 (65.0–68.1)	33.4 (31.9–35.0)	
25–34	6040	66.5 (65.3–67.7)	33.5 (32.3–34.7)	
35–44	5531	67.4 (66.1–68.6)	32.6 (31.4–33.9)	
45–54	5873	73.1 (72.0–74.2)	26.9 (25.8–28.0)	
55–67	4320	78.9 (77.7–80.1)	21.1 (19.9–22.3)	
Region				**
Capital Region of Denmark	7627	66.7 (65.6–67.7)	33.3 (32.3–34.4)	
Central Denmark Region	8524	70.8 (69.8–71.8)	29.2 (28.2–30.2)	
Zealand Region of Denmark	4851	73.4 (72.1–74.6)	26.6 (25.4–27.9)	
North Denmark Region	4255	72.7 (71.3–74.0)	27.3 (26.0–28.7)	
Place of upbringing				**
Farm with livestock	3871	78.8 (77.4–80.0)	21.3 (20.0–22.6)	
Farm without livestock	594	69.4 (65.5–72.9)	30.6 (27.1–34.5)	
Rural area	4698	71.3 (70.0–72.6)	28.7 (27.4–30.0)	
Small town	8222	69.0 (68.0–70.0)	31.0 (30.0–32.0)	
Suburb	4938	66.8 (65.4–68.1)	33.2 (31.9–34.6)	
Inner city	2842	67.4 (65.6–69.1)	32.6 (30.9–34.4)	
Months with nasal or eye symptoms in participants with ARC				
April–August	2330	9.53 (8.40–10.8)	90.5 (89.2–91.6)	**
September–March	40	20.0 (10.5–34.8)	80.0 (65.2–89.5)	
Always	858	18.9 (16.4–21.6)	81.1 (78.4–83.6)	
Age at first nasal or eye symptom in participants with ARC (years)				
Before 10	934	6.75 (5.77–9.37)	93.3 (91.5–94.7)	**
10–30	2103	12.0 (10.7–13.7)	88.0 (86.6–89.3)	
31–50	421	27.1 (22.7–31.8)	72.9 (68.5–76.9)	
After 50	29	48.3 (30.0–66.5)	51.7 (34.4–68.6)	
Time since last nasal or eye symptoms in participants withARC				
1 year or less	3066	12.1 (11.0–13.3)	87.9 (86.7–89.0)	NS
More than 1 year	422	17.1 (13.8–20.9)	82.9 (79.1–86.2)	

*Notes:* Results are presented as percentages with corresponding 95% confidence intervals (CI) and medians with interquartile ranges (IQR). Groups were compared by Kruskall‐Wallis test for non‐normally distributed data, and chi‐squared test for categorical data whenever all estimated counts were >5, and a 10^5^‐fold Monte Carlo simulated estimate for Fisher's exact test whenever estimated counts were ≤ 5. P‐values were Bonferroni corrected, **p* < 0.05; **p < 0.001; NS: non‐significant.

Abbreviations: ARC, allergic rhinitis (AR) and allergic conjunctivitis (AC); F, female; M, male; non‐sensitized, allergen‐specific immunoglobulin E (Phadiatop) <0.35 kU/L; sensitized, allergen‐specific immunoglobulin E (Phadiatop) ≥ 0.35 kU/L.

^a^ Compared with the total number of participants in the column. ^b^Compared with the total number of participants in the row.
