# Supplementary figures and images for: Combinations of self‐reported rhinitis, conjunctivitis, and asthma predicts IgE sensitization in more than 25,000 Danes
Source: Clin Transl Allergy. 2021 Mar 30;11(1):e12013. doi: 10.1002/clt2.12013 (PMC8099331; doi:10.1002/clt2.12013)

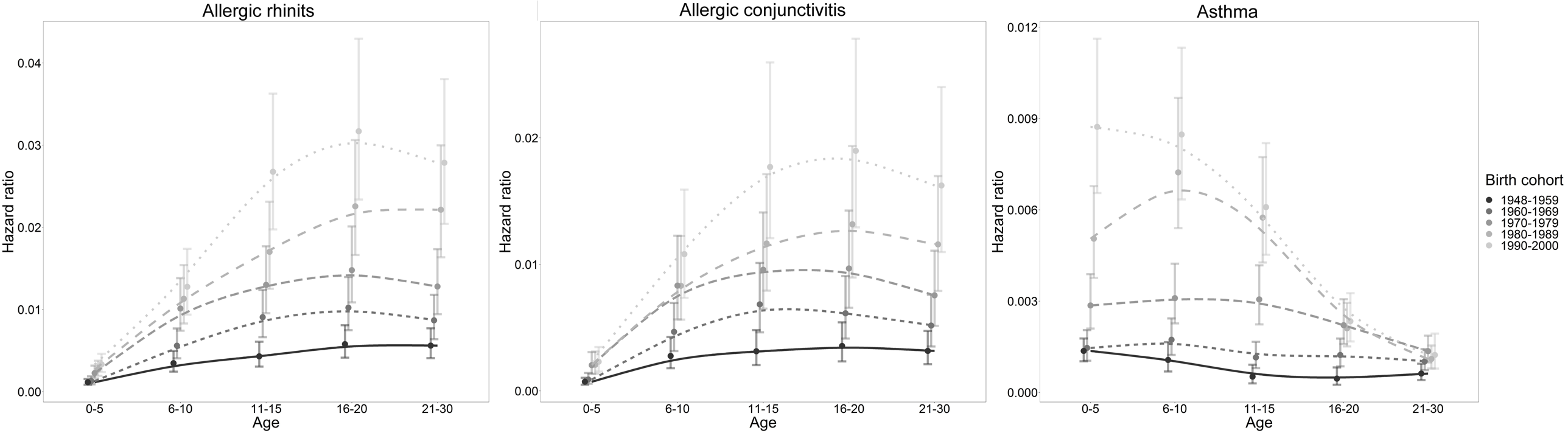

Supplement: Supplementary file 1 — Supplementary Material S1 [file CLT2-11-e12013-s001.jpg]
