# Supplementary material for: Combinations of self‐reported rhinitis, conjunctivitis, and asthma predicts IgE sensitization in more than 25,000 Danes
Source: Clin Transl Allergy. 2021 Mar 30;11(1):e12013. doi: 10.1002/clt2.12013 (PMC8099331; doi:10.1002/clt2.12013)
Supplement: Supplementary file 2 — Supplementary Material S2 [file CLT2-11-e12013-s002.docx]

Supporting Information

Article type: ORIGINAL RESEARCH

Mikkelsen S. et al

**Combinations of self-reported rhinitis, conjunctivitis, and asthma predicts IgE sensitization in more than 25,000 Danes**

Content

[Tables 2](#_Toc59201198)

[Table S1. Effect of the number of blood donations on the association between birth cohort and age at the time of first symptom of asthma, allergic rhinitis (nasal symptoms), or allergic conjunctivitis (eye symptoms) (N = 52,976) 2](#_Toc59201199)

[Table S2a. Risk factors for allergic rhino-conjunctivitis and asthma in female participants with Phadiatop measurements (N = 11,958) 3](#_Toc59201200)

[Table S2b. Risk factors for allergic rhino-conjunctivitis and asthma in male participants with Phadiatop measurements (N = 13,299) 4](#_Toc59201201)

[Table S3. Association between place of upbringing, and AR, AC, ARC, and asthma in all participants below 45 years (N = 32,145) 5](#_Toc59201202)

[Table S4. Association between place of upbringing, and ARC and inhalant allergen sensitization (regardless of asthma) (N = 25,257) 6](#_Toc59201203)

[Figure legend 7](#_Toc59201204)

[Figure S1. Association between birth and onset of symptoms 7](#_Toc59201205)

# Tables

| Table S1. Effect of the number of blood donations on the association between birth cohort and age at the time of first symptom of asthma, allergic rhinitis (nasal symptoms), or allergic conjunctivitis (eye symptoms) (N = 52,976) |
| --- |

| **Birth cohorts** | | | | | |
| --- | --- | --- | --- | --- | --- |
|  | **1948–1959** | **1960–1969** | **1970–1979** | **1980–1989** | **1990–2000** |
| Blood donations | N | N | N | N | N |
| 1–10 | 155 | 865 | 1,540 | 3,140 | 6,598 |
| 11–20 | 524 | 1,743 | 2,810 | 4,161 | 3,525 |
| >20 | 6,025 | 9,158 | 7,505 | 4,136 | 1,091 |
| ***Asthma symptoms*** | | | | | |
| Blood donations | HR (95% CI) | HR (95% CI) | HR (95% CI) | HR (95% CI) | HR (95% CI) |
| 1–10 | 1 | 1 | 1 | 1 | 1 |
| 11–20 | 0.94 (0.49–1.96) | 1.26 (0.94–1.72) | 0.91 (0.75–1.11) | **0.77 (0.67–0.88)** | 0.90 (0.80–1.01) |
| >20 | 0.57 (0.33–1.11) | 0.71 (0.55–0.95) | **0.69 (0.58–0.83)** | **0.72 (0.63–0.83)** | 1.03 (0.87–1.22) |
| ***Nasal symptoms*** | | | | | |
| Blood donations | HR (95% CI) | HR (95% CI) | HR (95% CI) | HR (95% CI) | HR (95% CI) |
| 1–10 | 1 | 1 | 1 | 1 | 1 |
| 11–20 | 1.15 (0.84–1.62) | 0.99 (0.87–1.14) | 1.12 (1.01–1.23) | 0.95 (0.88–1.02) | 0.97 (0.90–1.04) |
| >20 | 0.97 (0.73–1.33) | 0.92 (0.82–1.03) | 0.97 (0.89–1.06) | **0.87 (0.81–0.94)** | 0.89 (0.79–0.99) |
| ***Eye symptoms*** | | | | | |
| Blood donations | HR (95% CI) | HR (95% CI) | HR (95% CI) | HR (95% CI) | HR (95% CI) |
| 1–10 | 1 | 1 | 1 | 1 | 1 |
| 11–20 | 1.16 (0.79–1.74) | 0.99 (0.85–1.17) | 1.02 (0.91–1.15) | 0.99 (0.90–1.08) | 0.91 (0.84–0.99) |
| >20 | 0.86 (0.62–1.25) | 0.86 (0.75–0.99) | 0.98 (0.88–1.08) | **0.85 (0.77–0.93)** | 0.97 (0.85–1.10) |
| Distribution of participants between birth cohorts and number of blood donations at the date of inclusion in DBDS.  Poisson proportional hazard regression. Results are presented as hazard ratios (HR) with corresponding 95% confidence intervals (CI). The blood group 1–10 donations was reference. Adjusted for sex, smoking behavior (current, former or never smoker), parental allergy (yes/no), and BMI (continuous). Significant differences after Bonferroni correction are shown in bold. | | | | | |

| Table S2a. Risk factors for allergic rhino-conjunctivitis and asthma in female participants with Phadiatop measurements (N = 11,958) |
| --- |

|  | **Asthma (no AR, no AC)** | | **Asthma, and either AR or AC** | | **Asthma and ARC** | | **ARC (no asthma)** | | **Either AR or AC (no asthma)** | |
| --- | --- | --- | --- | --- | --- | --- | --- | --- | --- | --- |
|  | Crude | Adjusted | Crude | Adjusted | Crude | Adjusted | Crude | Adjusted | Crude | Adjusted |
| Predictors | RRR (95% CI) | RRR (95% CI) | RRR (95% CI) | RRR (95% CI) | RRR (95% CI) | RRR (95% CI) | RRR (95% CI) | RRR (95% CI) | RRR (95% CI) | RRR (95% CI) |
| Non-sensitized | 1 | 1 | 1 | 1 | 1 | 1 | 1 | 1 | 1 | 1 |
| Sensitized | **1.94**  **(1.57–2.41)** | **1.82**  **(1.47–2.27)** | **9.98**  **(7.14–14.0)** | **9.39**  **(6.69–13.2)** | **38.9**  **(28.7–52.9)** | **36.2**  **(26.5–49.4)** | **27.0**  **(23.2–31.5)** | **26.1**  **(22.3–30.6)** | **6.18**  **(5.23–7.30)** | **6.21**  **(5.23–7.38)** |
| No parental allergy | 1 | 1 | 1 | 1 | 1 | 1 | 1 | 1 | 1 | 1 |
| Parental allergy | **1.97**  **(1.59–2.42)** | **1.64**  **(1.32–2.03)** | **2.41**  **(1.67–3.48)** | **2.00**  **(1.36–2.92)** | **4.46**  **(3.55–5.60)** | **3.28**  **(2.55–4.23)** | **3.28**  **(2.86–3.75)** | **2.65**  **(2.25–3.12)** | **2.37**  **(1.97–2.86)** | **2.31**  **(1.89–2.82)** |
| Small municipality | 1 | 1 | 1 | 1 | 1 | 1 | 1 | 1 | 1 | 1 |
| Large municipality | 1.05  (0.88–1.22) | 0.86  (0.72–1.04) | 1.05  (0.75–1.46) | 0.91  (0.64–1.29) | **1.44**  **(1.16–1.79)** | 1.12  (0.88–1.42) | 1.09  (0.97–1.23) | 0.93  (0.80–1.07) | 0.88  (0.74–1.04) | 0.84  (0.70–1.01) |
| Non obese | 1 | 1 | 1 | 1 | 1 | 1 | 1 | 1 | 1 | 1 |
| Obesity | 1.34  (1.06–1.70) | 1.41  (1.11–1.79) | **2.11**  **(1.45–3.08)** | **2.36**  **(1.59–3.48)** | 1.12  (0.83–1.52) | 1.35  (0.97–1.88) | 0.93  (0.77–1.11) | 1.03  (0.84–1.27) | 1.04  (0.82–1.31) | 1.05  (0.83–1.34) |
| Never smoker | 1 | 1 | 1 | 1 | 1 | 1 | 1 | 1 | 1 | 1 |
| Current smoker | **1.46**  **(1.16–1.84)** | 1.37  (1.08–1.73) | 1.02  (0.63–1.65) | 1.05  (0.64–1.70) | 1.08  (0.79–1.47) | 1.10  (0.79–1.54) | 0.82  (0.68–0.99) | 0.88  (0.71–1.09) | 1.11  (0.88–1.40) | 1.12  (0.88–1.43) |
| Former smoker | 0.85  (0.67–1.06) | 1.02  (0.80–1.29) | 1.07  (0.73–1.57) | 1.27  (0.85–1.90) | 0.95  (0.73–1.24) | 1.24  (0.93–1.65) | 0.90  (0.78–1.05) | 1.09  (0.91–1.29) | 1.05  (0.87–1.28) | 1.11  (0.91–1.36) |
| > 10 blood donations | 1 | 1 | 1 | 1 | 1 | 1 | 1 | 1 | 1 | 1 |
| 1–10 blood donations | **1.97**  **(1.64–2.37)** | **1.50**  **(1.23–1.82)** | 1.49  (1.05–2.11) | 1.29  (0.88–1.88) | 1.50  (1.19–1.89) | 1.15  (0.88–1.49) | 1.17  (1.02–1.34) | 1.03  (0.87–1.22) | 0.82  (0.67–1.00) | 0.78  (0.63–0.97) |
| 45 years or older | 1 | 1 | 1 | 1 | 1 | 1 | 1 | 1 | 1 | 1 |
| Age below 45 years | **2.47**  **(2.00–3.06)** | **2.08**  **(1.64–2.63)** | **1.82**  **(1.26–2.62)** | 1.46  (0.97–2.20) | **1.99**  **(1.55–2.54)** | 1.26  (0.94–1.68) | **1.27**  **(1.12–1.45)** | 0.94  (0.80–1.11) | 0.92  (0.78–1.08) | 0.87  (0.72–1.06) |
| Multinomial logistic regression analysis Results are presented as relative risk ratios (RRR) with 95% confidence intervals (CI). Crude: no adjustment. Adjusted: adjustment for Phadiatop, parental allergy, current municipality, obesity, smoking behavior, total number of blood donations, and age. Controls served as base. Significant results after Bonferroni correction are shown in bold.  Sensitized: allergen-specific immunoglobulin E (Phadiatop) ≥ 0.35 kU/l. Non-sensitized: allergen-specific immunoglobulin E (Phadiatop) < 0.35 kU/l. AR: allergic rhinitis. AR: allergic conjunctivitis. ARC: both AR and AC. | | | | | | | | | | |

| Table S2b. Risk factors for allergic rhino-conjunctivitis and asthma in male participants with Phadiatop measurements (N = 13,299) |
| --- |

|  | **Asthma (no AR, no AC)** | | **Asthma, and either AR or AC** | | **Asthma and ARC** | | **ARC (no asthma)** | | **Either AR or AC (no asthma)** | |
| --- | --- | --- | --- | --- | --- | --- | --- | --- | --- | --- |
|  | Crude | Adjusted | Crude | Adjusted | Crude | Adjusted | Crude | Adjusted | Crude | Adjusted |
| Predictors | RRR (95% CI) | RRR (95% CI) | RRR (95% CI) | RRR (95% CI) | RRR (95% CI) | RRR (95% CI) | RRR (95% CI) | RRR (95% CI) | RRR (95% CI) | RRR (95% CI) |
| Non-sensitized | 1 | 1 | 1 | 1 | 1 | 1 | 1 | 1 | 1 | 1 |
| Sensitized | **3.15**  **(2.63–3.77)** | **2.86**  **(2.38–3.44)** | **15.4**  **(10.5–22.5)** | **13.8**  **(9.39–20.4)** | **87.0**  **(56.0–135)** | **82.2**  **(52.2–129)** | **44.7**  **(37.1–53.9)** | **42.3**  **(35.0–51.0)** | **8.83**  **(7.51–10.4)** | **8.58**  **(7.27–10.1)** |
| No parental allergy | 1 | 1 | 1 | 1 | 1 | 1 | 1 | 1 | 1 | 1 |
| Parental allergy | **2.17**  **(1.73–2.72)** | **1.67**  **(1.32–2.11)** | **3.70**  **(2.55–5.37)** | **2.69**  **(1.82–3.96)** | **5.51**  **(4.47–6.81)** | **3.78**  **(2.98–4.78)** | **3.67**  **(3.22–4.19)** | **2.77**  **(2.35–3.26)** | **2.52**  **(2.08–3.06)** | **2.15**  **(1.74–2.64)** |
| Small municipality | 1 | 1 | 1 | 1 | 1 | 1 | 1 | 1 | 1 | 1 |
| Large municipality | **1.40**  **(1.17–1.67)** | 1.12  (0.93–1.35) | 1.33  (0.96–1.83) | 0.96  (0.68–1.35) | 1.27  (1.05–1.54) | 0.88  (0.71–1.09) | **1.26**  **(1.13–1.41)** | 0.98  (0.85–1.13) | 1.18  (1.01–1.37) | 1.08  (0.91–1.27) |
| Non obese | 1 | 1 | 1 | 1 | 1 | 1 | 1 | 1 | 1 | 1 |
| Obesity | 1.38  (1.09–1.76) | 1.47  (1.15–1.88) | 1.21  (0.77–1.90) | 1.33  (0.83–2.12) | 1.15  (0.87–1.52) | 1.30  (0.96–1.77) | 0.80  (0.67–0.96) | 0.86  (0.69–1.06) | 1.10  (0.89–1.38) | 1.14  (0.90–1.45) |
| Never smoker | 1 | 1 | 1 | 1 | 1 | 1 | 1 | 1 | 1 | 1 |
| Current smoker | 1.16  (0.90–1.49) | 1.09  (0.85–1.41) | 1.12  (0.71–1.75) | 1.05  (0.66–1.66) | 0.58  (0.41–0.81) | **0.54**  **(0.38–0.78)** | **0.67**  **(0.55–0.80)** | **0.63**  **(0.51–0.78)** | 0.72  (0.57–0.93) | 0.71  (0.55–0.92) |
| Former smoker | 0.94  (0.75–1.16) | 1.08  (0.86–1.35) | 0.76  (0.50–1.15) | 0.92  (0.60–1.42) | 0.70  (0.54–0.89) | 0.88  (0.67–1.15) | **0.72**  **(0.63–0.83)** | 0.86  (0.72–1.01) | 0.88  (0.73–1.06) | 0.96  (0.79–1.17) |
| > 10 blood donations | 1 | 1 | 1 | 1 | 1 | 1 | 1 | 1 | 1 | 1 |
| 1–10 blood donations | **1.92**  **(1.56–2.36)** | **1.46**  **(1.17–1.82)** | 1.66  (1.13–2.45) | 1.17  (0.77–1.78) | **1.83**  **(1.46–2.31)** | 1.33  (1.02–1.73) | 1.24  (1.07–1.43) | 0.99  (0.83–1.19) | 1.03  (0.83–1.27) | 0.93  (0.74–1.18) |
| 45 years or older | 1 | 1 | 1 | 1 | 1 | 1 | 1 | 1 | 1 | 1 |
| Age below 45 years | **2.21**  **(1.81–2.69)** | **1.72**  **(1.38–2.14)** | **2.14**  **(1.49–3.06)** | 1.40  (0.94–2.08) | **2.07**  **(1.67–2.57)** | 1.19  (0.93–1.53) | **1.44**  **(1.28–1.61)** | 0.93  (0.80–1.08) | 1.04  (0.90–1.21) | 0.79  (0.66–0.95) |
| Multinomial logistic regression analysis Results are presented as relative risk ratios (RRR) with 95% confidence intervals (CI). Crude: no adjustment. Adjusted: adjustment for Phadiatop, parental allergy, current municipality, obesity, smoking behavior, total number of blood donations, and age. Controls served as base. Significant results after Bonferroni correction are shown in bold.  Sensitized: allergen-specific immunoglobulin E (Phadiatop) ≥ 0.35 kU/l. Non-sensitized: allergen-specific immunoglobulin E (Phadiatop) < 0.35 kU/l. AR: allergic rhinitis. AR: allergic conjunctivitis. ARC: both AR and AC. | | | | | | | | | | |

| Table S3. Association between place of upbringing, and AR, AC, ARC, and asthma in all participants below 45 years (N = 32,145) | | | | | |
| --- | --- | --- | --- | --- | --- |
|  | Asthma (no AR, no AC)  (N = 1,813) | Asthma, and either AR or AC  (N = 494) | Asthma and ARC  (N = 1,269) | ARC (no asthma)  (N = 3,663) | Either AR or AC (no asthma)  (N = 1,682) |
| Place of upbringing | RRR (95% CI) | RRR (95% CI) | RRR (95% CI) | RRR (95% CI) | RRR (95% CI) |
| Farm with livestock | 1 | 1 | 1 | 1 | 1 |
| Farm without livestock | 0.95 (0.68–1.34) | 0.52 (0.21–1.29) | 1.23 (0.79–1.92) | **1.55 (1.19–2.02)** | 1.31 (0.94–1.81) |
| Rural area | 0.99 (0.83–1.17) | 0.98 (0.68–1.39) | 1.22 (0.96–1.55) | **1.53 (1.31–1.77)** | 1.09 (0.90–1.31) |
| Small town | 0.93 (0.80–1.09) | 1.37 (1.00–1.88) | **1.48 (1.19–1.84)** | **1.61 (1.40–1.85)** | 1.05 (0.89–1.24) |
| Suburb of city | 1.00 (0.84–1.19) | 1.23 (0.87–1.73) | **1.47 (1.17–1.86)** | **1.67 (1.44–1.94)** | 0.95 (0.79–1.14) |
| Inner city | 0.99 (0.81–1.21) | 0.91 (0.60–1.39) | **1.58 (1.22–2.04)** | **1.64 (1.39–1.93)** | 1.00 (0.80–1.24) |
| Multinomial logistic regression analysis. Results are presented as relative risk ratios (RRR) with corresponding 95% confidence intervals (CI). Adjusted for sex, age (ten-year strata), smoking behavior (current, former or never smoker), parental allergy (yes/no), BMI (continuous), current municipality residence (small/large), and total number of blood donations (1–10, 11–20, > 20). Controls served as base. Farm with livestock was reference. Significant results after Bonferroni correction are shown in bold.  AR: allergic rhinitis. AR: allergic conjunctivitis. ARC: both AR and AC. | | | | | |

| Table S4. Association between place of upbringing, and ARC and inhalant allergen sensitization (regardless of asthma) (N = 25,257) | | | |
| --- | --- | --- | --- |
|  | Sensitized  without ARC  (N = 4,401) | Sensitized  with ARC  (N = 3,052) | Non-sensitized  with ARC  (N = 443) |
| Place of upbringing | RRR (95% CI) | RRR (95% CI) | RRR (95% CI) |
| Farm with livestock | 1 | 1 | 1 |
| Farm without livestock | 1.25 (0.99–1.58) | **1.87 (1.40–2.50)** | 0.64 (0.29–1.40) |
| Rural area | 1.17 (1.04–1.33) | **1.71 (1.45–2.02)** | 0.88 (0.63–1.22) |
| Small town | **1.24 (1.11–1.39)** | **1.96 (1.68–2.28)** | 0.81 (0.60–1.09) |
| Suburb of city | **1.31 (1.16–1.48)** | **2.11 (1.80–2.47)** | 0.93 (0.68–1.28) |
| Inner city | **1.31 (1.15–1.51)** | **2.15 (1.80–2.56)** | 1.11 (0.78–1.58) |
| Multinomial logistic regression analysis. Results are presented as relative risk ratios (RRR) with corresponding 95% confidence intervals (CI). Adjusted for sex, age (ten-year strata), smoking behavior (current, former or never smoker), parental allergy (yeas/no), BMI (continuous), current municipality residence (small/large), and total number of blood donations ((1–10, 11–20, > 20). Non-sensitized without ARC served as base. Farm with livestock was reference. Significant results after Bonferroni correction are shown in bold.  Sensitized: allergen-specific immunoglobulin E (Phadiatop) ≥ 0.35 kU/l. Non-sensitized: allergen-specific immunoglobulin E (Phadiatop) < 0.35 kU/l. ARC: both allergic rhinitis and allergic conjunctivitis. | | | |

# Figure legend

### Figure S1. Association between birth and onset of symptoms

Association between birth cohorts and the age at the time of first symptom of self-reported asthma, allergic rhinitis, and allergic conjunctivitis. Smoothed hazard ratios with corresponding 95% confidence intervals. Adjusted by number of blood donations, sex, smoking status, parental allergy, and BMI. Participants can appear in more than one symptom group.
